# The Mental and the Physical

**Published:** 1870-02

**Authors:** F. C. Marsh

**Affiliations:** Mount Pleasant, Iowa


### CHICAGO MEDICAL EXAMINER.

N. S. DAVIS, M.D., Editor.

VOL. XI. FEBRUARY, 1870. NO. 2.

### Original Contributions.

### ARTICLE V.

### THE MENTAL AND THE PHYSICAL.

By F. C. MARSH., M.D., Mount Pleasant, Iowa.

He who fearlessly thinks, independent of the influence of received opinion, and presents those thoughts to his fellow-men, undisguised and ungarbled, dares much; for prejudice is a tidal, which nothing save the firm shores of established truth can turn back; and to change the flow of thoughts from accustomed channels is like making a new bed for a great stream, whose waters have coursed along the broad old way for centuries.

Yet men are allowed a certain latitude in which to theorize, nay dream day dreams, and repeat them to their fellow-men. Sometimes these waking visions savor of truth. How know we in metaphysics, which are the dreams and which the realities? Surely it were hard to tell!

The peculiar relation existing between the physical and the mental, and the bearing of the one upon the other, has been the subject of theory and investigation, since man began to think of himself. We gather nothing wholly satisfactory from the multiplicity of theories and speculations of the metaphysical works, thus far produced; simply arbitrarily drawn divisions of mind, into the external and internal, and these again into subdivisions. All the differences we find in the peculiarities of men are attributed to their different mentalities; while nothing is said of their physical organisms, and the bearing which these must have on all mental manifestations.

I hold it as a truth, almost self-evident to every investigating man, that every thought, every emotion, every conception, has its origin in some suggestion from without, which comes to the mind through the media of the physical. If this be true, how much of the almost infinite difference we find in men can be satisfactorily explained, by carefully studying the minute structure of these compound beings.

Differences in color, size, expression, and form of the eyes of the several persons we meet, is a fact patent; yet we never realize that these variances modify the impressions received by them and transmitted to the brain, through the media of nerves as variable as the external appearances of the organs receiving those impressions: the arrangement of nerve fibre and the quality of that fibre must be elements influencing the chain of transmitted impressions.

The influence of the different modifications of the lens, upon transmitted rays of light, is a fact too familiar to demand mention; yet in these eyes we have all the modifications of the lens, in all degrees of perfection; but who of us give our fellow-men, who cannot perceive the beautiful as readily as we, credit for possessing as much capacity for such perception, and only only faulty in the possession of an imperfect instrument, through which to behold God's art gallery? Is mind (a scintellation from Infinite perfection) at fault for not appreciating that which is never brought within the range of its observation? Again, we hear sounds, and these sounds, when properly combined, become harmonious and musical: all do not have the same impressions conveyed to the mind of these sounds; one catches all the minuteness of change and delicacy of tone of the ceaseless motion around, while another only hears the collective rush of the combined motion—and why? Simply, there is in the one a delicate tympanum, and the chain of bones, conveyers of sound from the tympanum to the internal ear, are morbid, and the internal ear is delicately formed, and, beyond this, the arrangement of nerve fibre is that most propitious for the transmission of impressions; while, in the other, one or more of these conditions, essential to the full appreciation and perception of melody, is wanting. Mind has no part in this, for mind has had no opportunity to receive, in the second case, what mind did in the first. Touch, another media, through which mind receives impressions from the without, is dependant upon (in a great measure) the thinness of the epidermis, or outer skin, which covers in the papillæ, or essential element of touch. If this epidermis be thick and horny, as in the hand of the laborer at manual toil, of necessity the impressions of texture and peculiarities of substances, as received through this media, must be less correct and definite, than those brought to the cognizance of the mind, by the delicately-fingered lady's organism.

Taste and smell are subject to the same laws.

We speak of the expression of countenance as being an index of the mental characteristics of the possessor, and assume that these characteristics are the moulders of that expression. This is true, just in the measure we admit that these peculiarities of mental manifestations are the result, directly, of the peculiar physical organism with which that mind is associated; for feature and expression are the title-pages of our physical beings, and only thus, indirectly, of our mentality; for all the mind's pabulum comes through this physical, and all its evolutions are made manifest again through the same channel, and receives its impress in this transmission. But, independent of this subserviency, mind is broader, deeper, and mightier than can be realized, so long as it wears the fetters of the physical, and has no index whatever as to its real peculiarities in that physical.

As the brain is the headquarters of the vast telegraphic company of impressions, and as each nerve has a peculiar class of impressions to transmit, and as these nerves severally, in pairs (the cranial nerves, I mean), are from different and distinct parts of the brain (thus proving that each portion of that brain has a peculiar office), and as brains are variable in size, shape, and texture, are we not justifiable in concluding, that from this peculiarity of organism, comes much of that which we term individuality? Can we expect men to be alike in apparent power of intellect, when the windows through which those intellects gather visions of the beauty, grandeur, and extent of the vast creation are so variable in size and character? Can we expect them to draw the same conclusions from things so differently seen? A slight pressure on some portion of a nerve, conveying an impression, would materially affect that impression, either to enhance or diminish it; and how often slight impairments in the regularity of the circulation, which may increase or diminish pressure, on a nerve contiguous to a vessel, occurs. And the flow of blood materially influences the action of the brain, increased in quantity and rapidity to a certain extent, and exhilaration and stimulation of brain action are the results; still further increased, and oppression obtains. Hence it is, that good talkers are men who need the stimulus of an audience to quicken their life-current, in order to the highest brain-action. While good writers need no such stimulus, and when under such excitement have brain-action oppressed, by an overflow of blood to that organ. The sluggish, indolent, man, whose pulse beats slow, and whose breath comes in long drawn gusts, when angered, to-morrow, may become quick in thought, and the volley of invective and sarcasm which flows from him will astonish all. Great danger may make him a miracle of invention, daring, and heroism. And these excitants quicken pulse and breath. There are no two individuals physically alike—face and form give personality to each—and there is almost as much dissimilarity in their anatomical structure. Changes in distribution of nerves and arteries are ever met with in the dissecting-room; the same general type of course is followed, but minutiæ is ever variable. Susceptibility to pain varies vastly in different individuals; and this variance is but an example of the degrees of facility of transmission of impressions, in these parties. Mind is not the controller of this, nor, indeed, is it at all responsible for it, but it is simply dependent on the organism of the neural system.

Our surroundings, as to temperature, purity of atmosphere, character of food, and necessities for action, are influences of mental manifestations. Under the debilitating influence of a high temperature, how difficult it is to induce brain or muscular action, such as would be almost involuntary in the lower temperatures, unless these be so severe as to overpower us. Who can think in an atmosphere of carbonic acid gas? and the ill-fed being has no higher aim than the attainment of that which will satiate his ever-knawing hunger.

If there had been no necessity for action—if food, water, and clothing had been supplied to the "*genus homo*," as to plants, what discoveries, what inventions, what language, would then have been? Verily (I believe), none! And, yet, can we say that mind is amenable to temperature, that it is affected by carbonic acid, that it becomes hungry, and needs clothing? And if it be not amenable to these influences, why make it responsible for all those imperfections which are simply the results of an imperfected physical mechanism. Steam is the same power, whether applied to the engine of perfect construction or to one of improper structure, though the manifestations of that power are vastly different. Mind is, I believe, a constant quantity, a spiritual essence, given to all rational beings, in the same measure; and, as I have tried to prove, in the past pages, influenced, so far as its manifestations are concerned, by its entire dependence on the physical, for all intimations of the created universe; and by these more or less perfect impressions, individualized and limited in capability.

With these views of human life before us, dare we critise our fellow-men, condemning their opinions as false and them as dishonest, for entertaining such views? Shall we speak slightingly of who is inappreciative of music, art, and the beauty of nature and has no conception of the grandeur of the world around him? Dare we make light of the poor, "half-witted" being, whose greatest fault is, that that organism, through which mind is made manifest, is signally imperfect, so that no food, no sunlight, no warmth, comes to the imprisoned spirit there; and it hibernates through the long winter of its confinement here, returning to the God who gave it, an infant, yet to develop into the manhood of soul-growth, after is liberation from the trammels that now starve it? With this belief, will we not find charity more easily exercised, God's goodness more discernible, and be better content with life and our fellow-men? With education, all our physical being becomes more nearly perfect, and, of necessity, our mental manifestations keep pace with the character of culture we give our physical. Sight, hearing, touch, taste, and smell will be the avenues through which beauty or deformity will be perceived by our immortal part; and as we exercise these in the direction of the beautiful in nature, and in thought, so will our mental manifestations partake of the beautiful and good.

